# Association between tumor-infiltrating lymphocytes and oncotype DX estrogen receptor, progesterone receptor, and human epidermal growth factor receptor 2 single gene scores in hormone receptor-positive/HER2-negative breast cancer

**DOI:** 10.3389/fonc.2026.1677929

**Published:** 2026-01-27

**Authors:** Masahiro Ohara, Emi Mikami, Wakako Inohana, Taeko Kurosawa, Ayako Nakame, Ayaka Sakakibara, Yuki Ichinose, Akihiro Fujimoto, Asami Nukui, Aya Asano, Hiroko Shimada, Kyoko Asai, Masataka Hirasaki, Hideki Yokogawa, Kazuo Matsuura, Hiroshi Ishiguro, Takahiro Hasebe, Nobuko Fujiuchi, Akihiko Osaki, Toshiaki Saeki

**Affiliations:** 1 Department of Breast Oncology, Saitama Medical University International Medical Center, Hidaka, Saitama, Japan; 2 Department of Breast Oncology, Saitama Medical University Hospital, Moroyama, Saitama, Japan; 3 Department of Clinical Cancer Genomics, Saitama Medical University International Medical Center, Hidaka, Saitama, Japan

**Keywords:** breast cancer, hormone receptor-positive/humanepidermal growth factor receptor 2-negative breast cancer, oncotype Dx, RT-PCR, tumor-infiltrating lymphocytes

## Abstract

**Introduction:**

Tumor-infiltrating lymphocytes (TILs) are established biomarkers in triple-negative and human epidermal growth factor receptor 2 (HER2)-positive breast cancers; however, their clinical significance in hormone receptor-positive, HER2-negative (HR+/HER2−) breast cancer remains unclear. This study aimed to investigate the association between TILs and Oncotype DX single gene scores for estrogen receptor (ER), progesterone receptor (PgR), and HER2 in HR+/HER2− breast cancer.

**Methods:**

We retrospectively analyzed 260 patients with HR+/HER2− breast cancer who underwent surgery and Oncotype DX testing at Saitama Medical University International Medical Center between January 2022 and October 2024. TILs were evaluated on hematoxylin and eosin–stained slides according to the International TILs Working Group 2014 guidelines, with high TILs defined as ≥10%. Associations between TILs, clinicopathological factors, and Oncotype DX single gene scores were examined using statistical analyses, including logistic regression. Additionally, publicly available data from The Cancer Genome Atlas (TCGA) cohort were analyzed for validation.

**Results:**

High TIL levels were observed in 32 cases (12.3%). Tumors with high TILs showed significantly lower Oncotype DX single gene expression of ER (9.8 ± 1.9 vs. 10.5 ± 1.3, p < 0.01), PgR (6.3 ± 2.2 vs. 7.4 ± 1.8, p < 0.01), and HER2 (8.5 ± 0.7 vs. 9.2 ± 0.6, p < 0.001) compared with tumors with low TILs. Multivariate analysis identified node-negative status (odds ratio [OR]: 0.266; p = 0.0159) and lower HER2 single gene expression (OR: 0.293; p = 0.00144) as independent predictors of high TILs. TCGA analysis confirmed that lower HER2 mRNA expression was associated with increased chemokine gene expression.

**Discussion:**

In HR+/HER2− breast cancer, tumors with lower HER2 mRNA expression exhibit higher lymphocytic infiltration, suggesting the presence of a distinct immunologically active subset. Oncotype DX single gene scores, particularly HER2, may provide information beyond recurrence risk prediction and help identify patients who may benefit from immune-modulating therapeutic strategies.

## 1 Introduction

Tumor-infiltrating lymphocytes (TILs) are crucial biomarkers for breast cancer, reflecting the host immune response against tumor cells. In triple-negative breast cancer (TNBC) and human epidermal growth factor receptor 2 (HER2)-positive subtypes, high TIL density is associated with an enhanced prognosis and greater response to adjuvant or neoadjuvant chemotherapy (1–3). Therefore, TIL assessment has been incorporated into the clinical practice guidelines for these subtypes (4). However, the clinical significance and biological role of TILs in hormone receptor (HR)-positive and HER2-negative breast cancer remain unclear because of their generally lower abundance in this group (5, 6).

For HR-positive and HER2-negative early breast cancer, the Oncotype DX assay is widely used to guide treatment decisions. It is a clinically validated multigene assay that uses reverse transcription polymerase chain reaction (RT-PCR) to measure the expression of 21 genes in formalin-fixed, paraffin-embedded tumor tissue. Based on the results of large prospective trials, including TAILORx and RxPONDER, the Oncotype DX recurrence score (RS) serves as both a prognostic tool for distant recurrence risk and predictive biomarker for chemotherapy benefit in selected patients (7–9).

Although immunohistochemistry (IHC) is routinely used to assess estrogen receptor (ER), progesterone receptor (PgR), and HER2 expression, we hypothesized that mRNA-based quantification using Oncotype DX single gene scores may better reflect TIL infiltration-associated biological diversity. Therefore, this study aimed to assess whether Oncotype DX single gene scores for ER, PgR, and HER2 are associated with TIL extent in ER-positive and HER2-negative breast cancer.

## 2 Patients and methods

### 2.1 Patients

In this retrospective study, we analyzed data from patients who underwent surgery in our department between January 2022 and October 2024. The study was approved by the Institutional Review Board of Saitama Medical University (approval number: 20-105), with waived informed consent because of its retrospective nature. The tumors were classified according to the pathological Union for International Cancer Control TNM classification (10). For histopathological examination, the surgically resected specimens were fixed in 10% formalin. The primary clinicopathological factors were assessed (**Table 1**). ER and PR statuses were determined based on the American Society of Oncology/College of American Pathologists (ASCO/CAP) guideline. Additionally, HER2 expression was categorized following the ASCO/CAP guidelines (11–13).

**Table 1:** Baseline clinicopathological characteristics of the study cohort (n = 260).

Variable	Median age (years)/No. of patients	%	Variable	No. of patients	%
Age (range)	56 (29–79)		ER status (%)		
Menopausal status			0	0	0
Premenopausal	108	41.5	1–9	2	0.8
Postmenopausal	152	58.5	10–50	5	1.9
Pathological tumor status			51–100	253	97.3
T1a	1	0.4	PgR status (%)		
T1b	13	5.0	0	16	6.2
T1c	71	27.3	1–9	6	2.3
T2	150	57.7	10–50	38	14.6
T3–T4	25	9.5	51–100	200	76.9
Pathological lymph node status			HER2 status		
pN0	161	61.9	0	28	10.8
pN1	99	38.1	1+	137	52.7
Histological type			2+ FISH−	95	36.5
Invasive ductal carcinoma	223	85.8	Ki67 labeling index		
Invasive lobular carcinoma	13	5.0	Median (Range)	32.0 (0–89.0)	
Others	24	9.2	Recurrence score		
Histological grade			0–10	67	25.8
Grade 1	70	26.9	11–25	136	52.3
Grade 2	150	57.7	26–100	57	21.9
Grade 3	39	15.0			
Unknown	1	0.4			

ER, estrogen receptor; PgR, progesterone receptor; HER2, human epidermal growth factor receptor 2.

Eligible patients had histologically confirmed HR-positive and HER2-negative breast cancer, with HER2 status documented by IHC as 0, + 1, or +2/*in situ* hybridization (ISH)-negative. Only patients who completed their initial surgical treatment and underwent Oncotype DX^®^ testing with a full report available, including single gene scores, were included. All patients were treatment-naïve at the time of surgery and had not received any neoadjuvant systemic therapy or radiotherapy prior to tumor sampling. Patients were excluded if they had a history of breast cancer and were receiving or had recently completed adjuvant hormonal therapy (within the past year) or if they had received neoadjuvant chemotherapy—because they were not considered suitable candidates for Oncotype DX^®^ testing.

**Figure 1:**
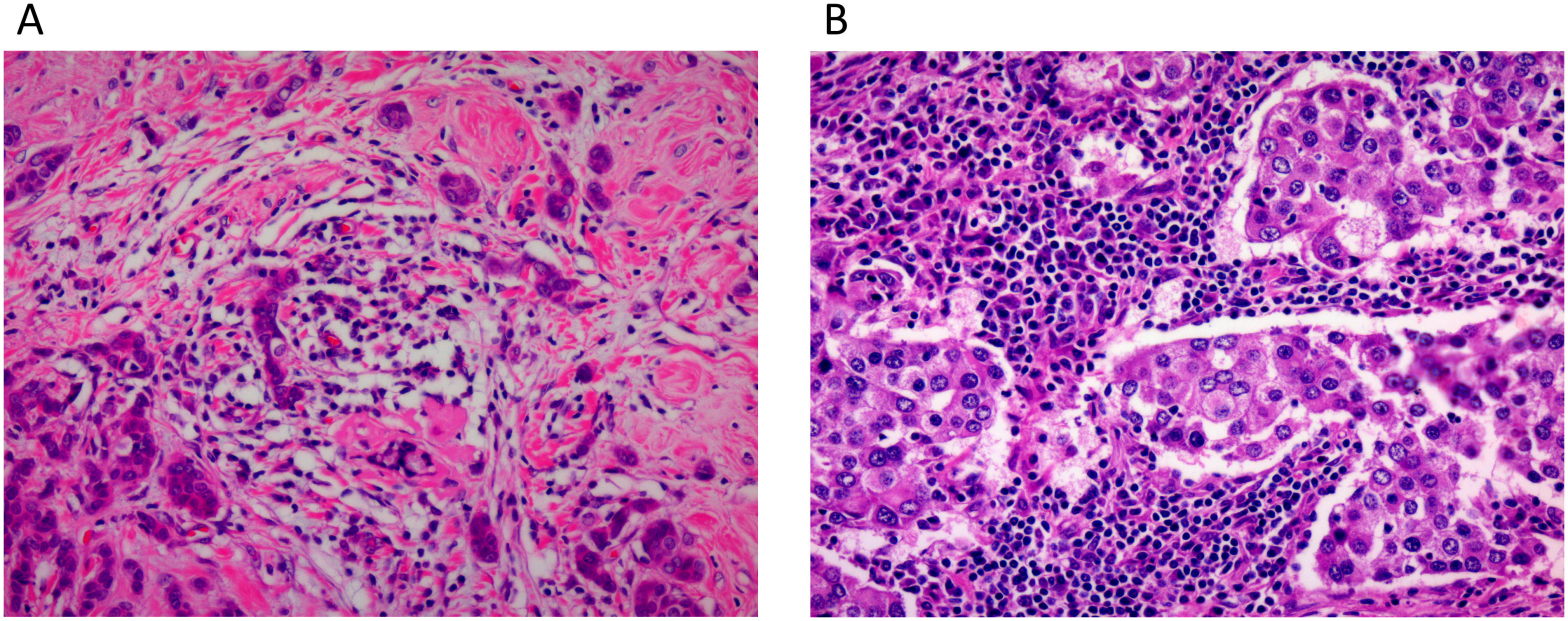
Representative H&E-stained images showing low and high tumor-infiltrating lymphocyte levels. Representative hematoxylin and eosin–stained images (original magnification ×200) showing low (**A**; TILs 1–9%) and high (**B**; TILs 10–100%) levels of tumor-infiltrating lymphocytes, assessed according to the International TILs Working Group 2014 guidelines.

TILs were assessed on hematoxylin and eosin (H&E)-stained slides, following the International TILs Working Group 2014 guidelines (4). The percentage of stromal area occupied by lymphocytes (TILs %) was assessed. Representative H&E-stained images demonstrating low and high TIL levels are shown in **Figure 1**.

### 2.2 TCGA RNA sequencing data analysis

To explore the molecular features associated with varying TILs levels, we analyzed publicly available RNA-seq data from the Breast Invasive Carcinoma (TCGA, Cell 2015) cohort using cBioPortal (https://www.cbioportal.org/). RNA-seq expression levels were quantified using RNA-seq by Expectation-Maximization (normalized values; unitless). We selected ER+/HER2- breast cancer cases (n = 444) based on clinical annotations and dichotomized them into high and low Erb-B2 receptor tyrosine kinase 2 (ERBB2) mRNA expression groups using the median log_2_(x+1) expression values. The sample IDs used in this analysis are listed in **Supplementary Table 1**. We then compared the expression of immune-related chemokines—including C-X-C motif chemokine ligand 9 (CXCL9), CXCL10, CXCL11, CXCL13, C-C motif chemokine ligand 5 (CCL5), CCL17, and CCL22—between the two groups using the Mann–Whitney U test. **Supplementary Table 1** lists the sample IDs extracted from the TCGA dataset that were included in our study.

These chemokines—known to recruit T cells, natural killer cells, and B cells into the tumor microenvironment (14–21)—were selected as representative lymphocyte-attracting factors that contribute to a more immunologically active phenotype.

### 2.3 Statistical analysis

Statistical analysis was performed using EZR (Easy R). The normality of the continuous variable distributions was assessed using the Kolmogorov–Smirnov test. If normality was confirmed, comparisons between the two groups were conducted using the t-test. For comparisons involving more than two groups, a one-way analysis of variance (ANOVA) was performed, and *post-hoc* multiple comparisons were conducted using the Bonferroni correction. Categorical variables were analyzed using the chi-square test or Fisher’s exact test as appropriate. Additionally, binary logistic regression analysis was performed to identify factors independently associated with high TILs, defined as ≥ 10% (4). Statistical significance was set at p < 0.05.

## 3 Results

**Table 1** summarizes the clinicopathological characteristics of the 260 Japanese women (aged 29–79 years; median age, 56 years) included in the study. The majority of tumors were classified as pathological T2–T4 stage (n = 175, 67.2%), with lymph node involvement in 99 patients (38.1%). Invasive ductal carcinoma accounted for 223 cases (85.8%), and 39 tumors (15.0%) were histological grade 3. All tumors exhibited ER expression ≥ 1% using IHC, with 254 tumors (97.3%) exhibiting high expression (51–100%). PgR expression was ≥ 1% in 244 tumors (93.8%) and high (51–100%) in 200 tumors (76.9%). HER2 IHC was negative (0 or 1+) in 165 tumors (63.5%), whereas 95 tumors (36.5%) were HER2 IHC 2+ and confirmed negative using ISH. The median Ki67 labeling index was 32.0% (range, 0–89%). Oncotype DX RS was available for all cases, where 67 (25.8%), 136 (52.3%), and 57 (21.9%) tumors were classified as low (0–10), intermediate (11–25), and high risk (26–100), respectively.

**Figure 2:**
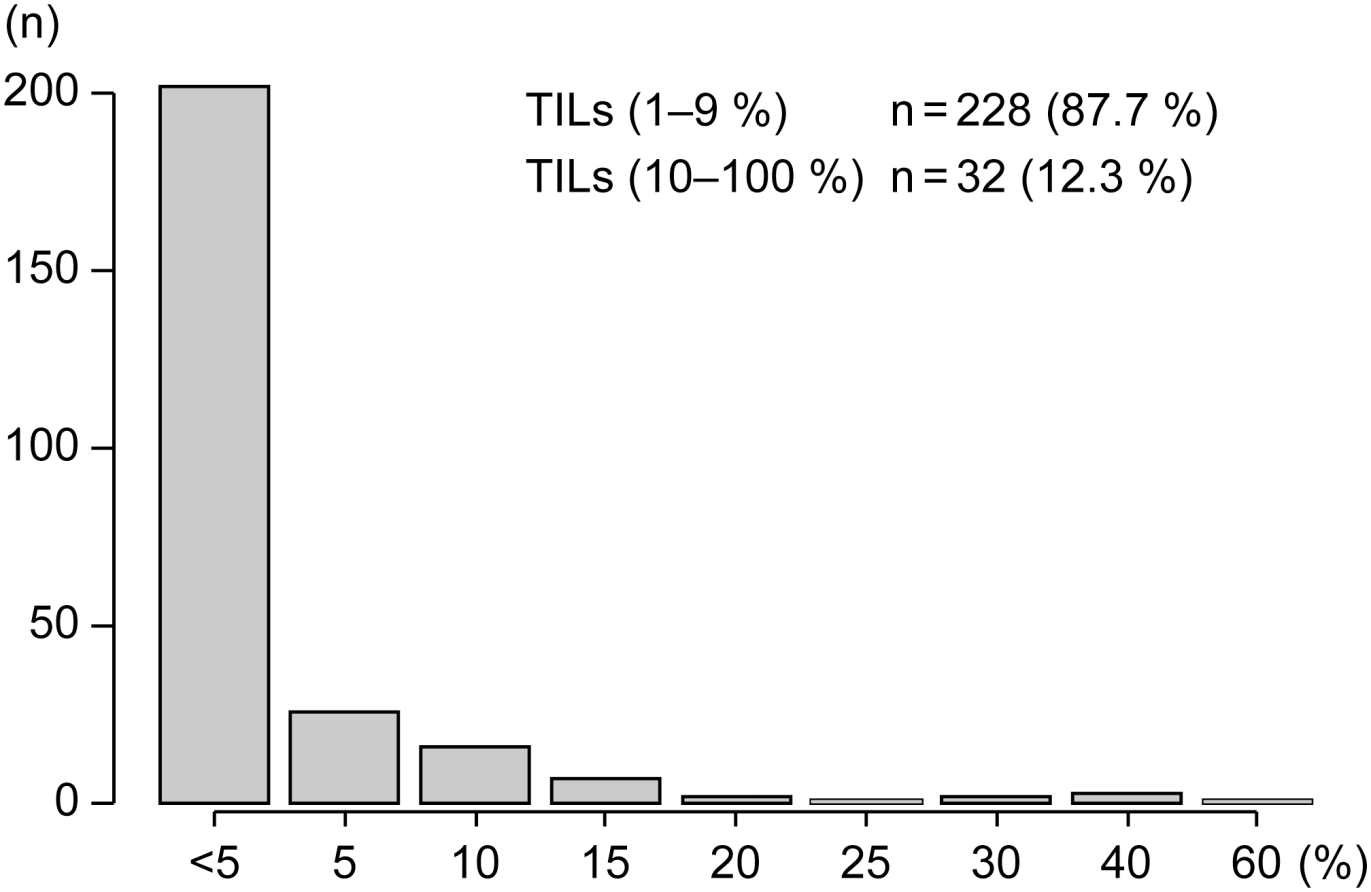
Frequency distribution of TILs scores in the study cohort. The x-axis indicates TILs scores (%), and the y-axis indicates the number of patients (n). TILs, tumor-infiltrating lymphocytes.

The distribution of TILs was predominantly low in this cohort, with 228 tumors (87.7%) exhibited TILs scores of 1–9%, whereas only 32 tumors (12.3%) exhibiting high TIL levels (10–100%). The frequency distribution is illustrated in **Figure 2**.

**Table 2** summarizes the associations between TIL levels and clinicopathological characteristics, including the RS. Tumors with high TIL levels were significantly associated with the absence of lymph node metastasis (p = 0.0189), higher histological grade (grade 3, p < 0.001), higher Ki67 labeling index (p < 0.001), and higher RS (p < 0.001). Other characteristics—age, menopausal status, tumor size, histological type, and ER, PgR, and HER2 expression by IHC—did not differ significantly between the low and high TIL groups (**Table 2**).

**Table 2:** Association between high tumor-infiltrating lymphocytes (TILs ≥ 10%) and clinicopathological characteristics.

Variable		TILs (1–9%)	TILs (10–100%)	P-value
Age (years)	Mean ± SD	56.0 ± 10.8	56.4 ± 11.0	0.832
Menopausal status				
	Premenopausal	97 (42.5%)	11 (34.4%)	0.446
	Postmenopausal	131 (57.5%)	21 (65.6%)	
Pathological T stage				
	T1	77 (33.8%)	8 (25.0%)	0.422
	T2–T4	151 (66.2%)	24 (75.0%)	
Pathological N stage				
	N0	135 (59.2%)	26 (81.2%)	0.0189
	N1	93 (40.8%)	6 (18.8%)	
Histological type				
	IDC	194 (85.1%)	28 (87.5%)	1.000
	ILC/Others	34 (14.9%)	4 (12.5%)	
Histological grade				
	Grade 1–2	201 (88.5%)	19 (59.4%)	< 0.001
	Grade 3	26 (11.5%)	13 (40.6%)	
Estrogen receptor status (%)				
	0–9	2 (0.9%)	0	1.000
	10–100	226 (99.1%)	32 (100%)	
Progesterone receptor status (%)				
	0–9	17 (7.5%)	5 (15.6%)	0.164
	10–100	211 (92.5%)	27 (84.4%)	
HER2 status				
	0	24 (10.5%)	4 (12.5%)	0.760
	1+, 2+, FISH−	204 (89.5%)	28 (87.5%)	
Ki67 labeling index (%)	Mean ± SD	32.0 ± 12.5	45.4 ± 18.1	< 0.001
Recurrence score	Mean ± SD	17.0 ± 10.4	28.7 ± 14.9	< 0.001

SD, standard deviation; IDC, invasive ductal carcinoma; ILC, invasive lobular carcinoma; HER2, human epidermal growth factor receptor 2.

**Figure 3:**
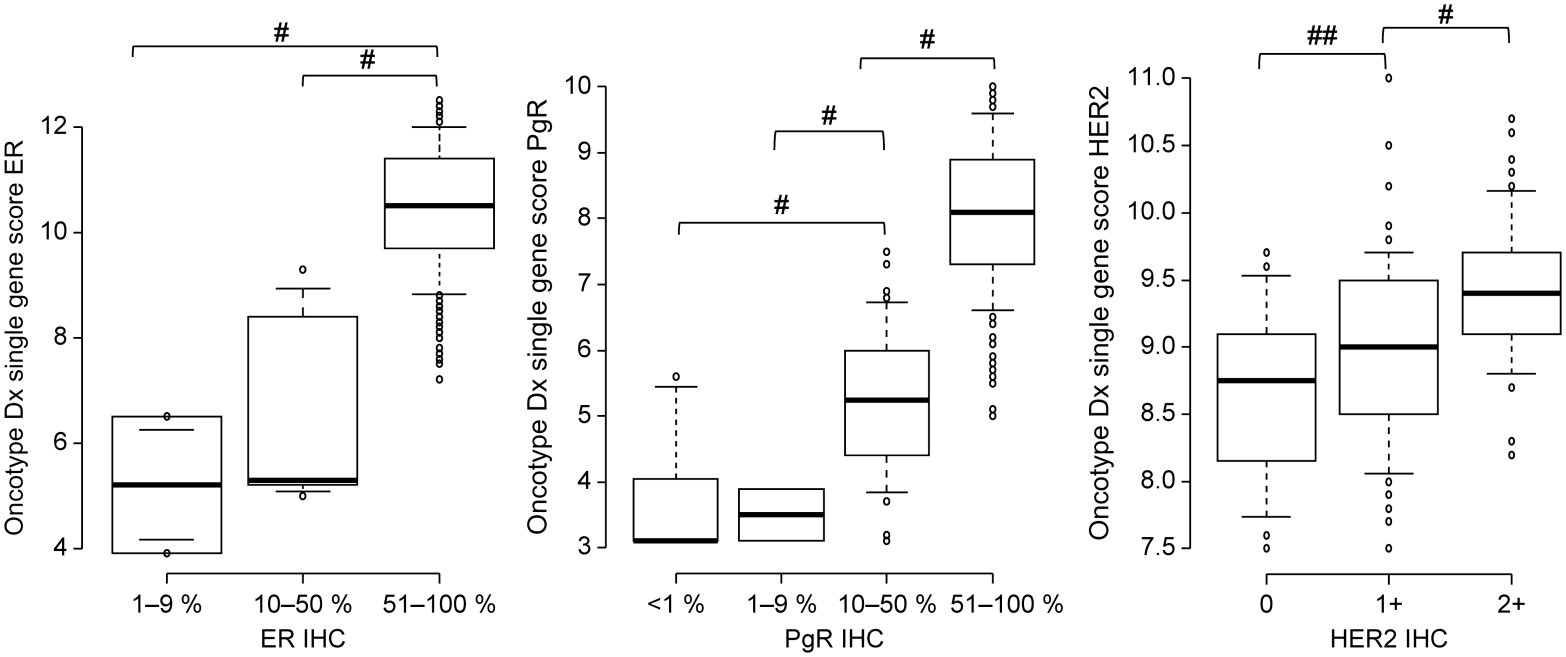
Oncotype DX single gene scores in relation to ER, PgR, and HER2 expression on immunohistochemistry. One-way ANOVA followed by Bonferroni correction is used to assess statistical significance. ER, estrogen receptor; PgR, progesterone receptor; HER2, human epidermal growth factor receptor 2; IHC, immunohistochemistry; ANOVA, analysis of variance. #p < 0.001 and ##p < 0.05 (Bonferroni correction).

The Oncotype DX single gene scores for ER, PgR, and HER2 were strongly associated with their corresponding IHC expression levels (**Figure 3**). ANOVA demonstrated significant differences among the IHC expression groups for ER, PgR, and HER2 (p < 0.001). For ER, the single gene score increased stepwise with IHC intensity, demonstrating significant differences between IHC 10–50% and 51–100% (p < 0.001), and between 1–9% and 51–100% (p < 0.001) but not between 1–9% and 10–50% (p = 0.48). For PgR, all pairwise comparisons among IHC 0, 1–9, 10–50, and 51–100% were statistically significant (p < 0.001). For HER2, the single gene score significantly increased stepwise from IHC 0 to 1+ (p < 0.001) and from 1+ to 2+ (p < 0.001).

To further assess the transcriptional features associated with immune infiltration, we compared the Oncotype DX single gene expression scores between tumors with low (1–9%) and high (10–100%) TIL levels. Tumors with high TIL levels exhibited significantly lower expression of ER (mean ± standard deviation: 9.8 ± 1.9 vs. 10.5 ± 1.3, p < 0.01), PgR (6.3 ± 2.2 vs. 7.4 ± 1.8, p < 0.01), and HER2 (8.5 ± 0.7 vs. 9.2 ± 0.6, p < 0.001) based on single gene scores than that of tumors with low TIL levels (**Table 3**). These results indicate that tumors with lower mRNA expression of ER, PgR, and HER2 exhibited higher TIL levels.

**Table 3:** Association between high tumor-infiltrating lymphocytes (TILs ≥ 10%) and Oncotype DX single gene scores.

Variable		TILs (1–9%)	TILs (10–100%)	P-value
Oncotype DX single gene score (ER)	Mean ± SD	10.5 ± 1.3	9.8 ± 1.9	< 0.01
Oncotype DX single gene score (PgR)	Mean ± SD	7.4 ± 1.8	6.3 ± 2.2	< 0.01
Oncotype DX single gene score (HER2)	Mean ± SD	9.2 ± 0.6	8.5 ± 0.7	< 0.001

SD, standard deviation; IDC, invasive ductal carcinoma; ILC, invasive lobular carcinoma; ER, estrogen receptor; PgR, progesterone receptor; HER2, human epidermal growth factor receptor 2.

To identify independent predictors of tumors with high TIL levels (≥ 10%), a multivariate logistic regression analysis was performed using clinicopathological factors and Oncotype DX single gene expression scores (**Table 4**). High TIL levels were independently associated with the absence of lymph node metastasis (odds ratio [OR]: 0.266; 95% confidence interval [CI]: 0.091–0.780; p = 0.0159) and lower HER2 single gene expression (OR: 0.293; 95% CI: 0.138–0.623; p = 0.00144). Other variables—menopausal status, tumor size, histological type, histological grade, ER and PgR single gene scores, and Ki67 labeling index—were not significantly associated with high TILs.

**Table 4:** Binary logistic regression analysis for predicting cases with high tumor-infiltrating lymphocytes (TILs ≥ 10%).

Factor	Category/Variable	Odds ratio	95% CI	P-value
Menopausal status	Premenopausal	0.564	0.188–1.690	0.307
Pathological tumor status	T2–T4	1.180	0.426–3.260	0.751
Pathological node status	N1	0.266	0.091–0.780	0.0159
Histological type	ILC, Others	0.475	0.126–1.800	0.273
Histological grade	Grade 3	2.000	0.680–5.900	0.208
ER status	Oncotype DX single gene score	0.766	0.554–1.060	0.105
PgR status	Oncotype DX single gene score	0.890	0.690–1.150	0.436
HER2 status	Oncotype DX single gene score	0.293	0.138–0.623	0.00144
Ki67 status	Ki67 labeling index (%)	4.380	0.107–179.000	0.436

ILC, invasive lobular carcinoma; ER, estrogen receptor; PgR, progesterone receptor; HER2, human epidermal growth factor receptor 2.

**Figure 4:**
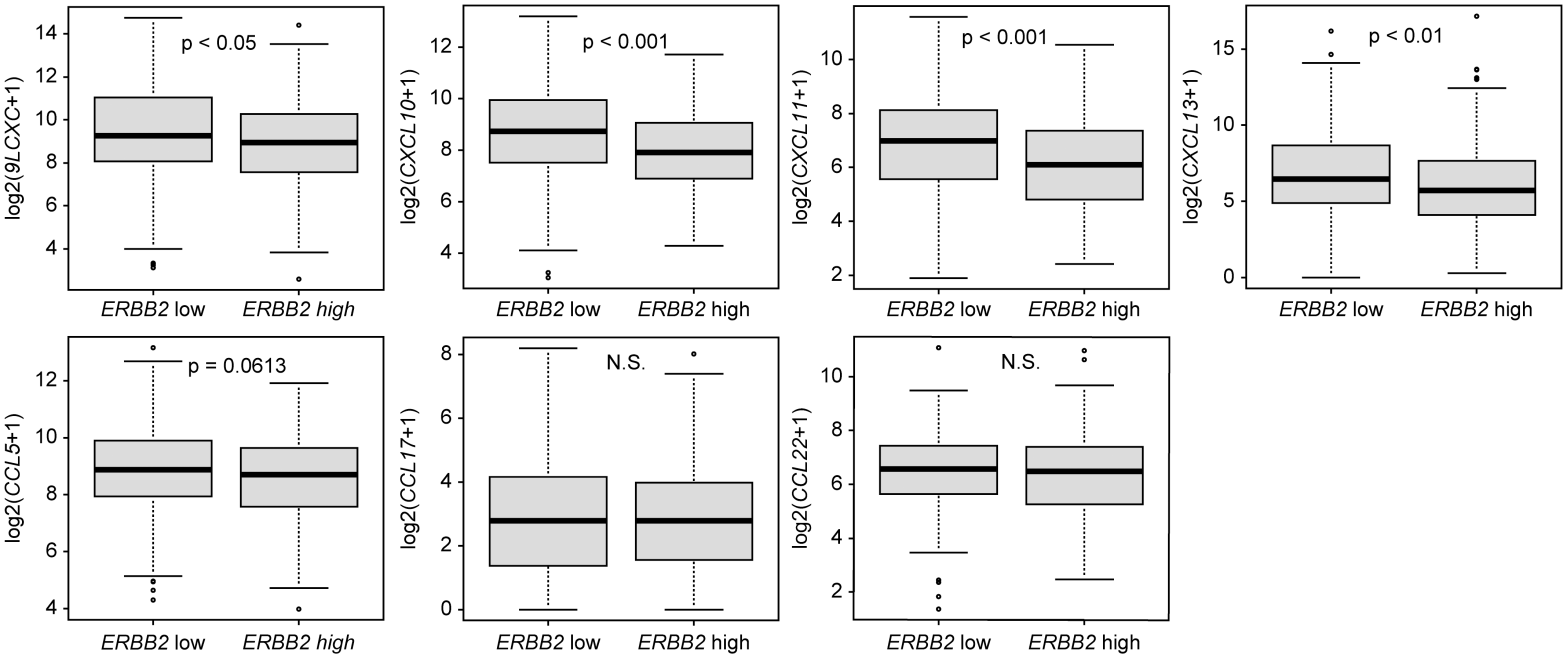
Comparison of immune-related chemokine expression levels between high and low ERBB2 mRNA expression groups in ER+/HER2- breast cancer cases (n = 444) from the TCGA cohort. Box plots demonstrate median, interquartile range, and range for each chemokine (CXCL9, CXCL10, CXCL11, CXCL13, CCL5, CCL17, and CCL22). Statistical significance is assessed using the Mann–Whitney U test. ERBB2, Erb-B2 receptor tyrosine kinase 2; ER, estrogen receptor; HER2, human epidermal growth factor receptor 2; TCGA, The Cancer Genome Atlas; CXCL, C-X-C motif chemokine ligand; CCL, C-C motif chemokine ligand; N.S., not significant.

In the TCGA dataset, tumors with lower ERBB2 mRNA expression exhibited significantly higher expression of multiple immune-related chemokines, including CXCL9, CXCL10, CXCL11, and CXCL13 (all p < 0.05). CCL5 expression trended to be higher (p = 0.0613), whereas CCL17 and CCL22 expression exhibited no significant differences (**Figure 4**).

## 4 Discussion

TILs have been extensively studied in TNBC and HER2-positive subtypes, where they are associated with a favorable prognosis and enhanced response to chemotherapy and immunotherapy (1–3). In contrast, the immunogenicity of HR-positive and HER2-negative (ER+/HER2-) breast cancers is generally considered limited. However, a subset of ER+/HER2- tumors can exhibit dense lymphocytic infiltration, similar to that observed in TNBC (22, 23). Notably, in ER+/HER2- tumors, higher TIL levels have not been consistently associated with enhanced prognosis. Paradoxically, numerous studies have reported shorter survival times in this subgroup (1).

Recent phase III trials—CheckMate 7FL and KEYNOTE-756—highlight the potential of immune checkpoint inhibitors in high-risk, early-stage ER+/HER2- breast cancer, although the observed clinical benefits have been largely restricted to increased pathological complete response (pCR) rates (24, 25). In the CheckMate 7FL study, nivolumab addition enhanced pCR, even in tumors with low but measurable stromal TILs (sTILs > 1%). Although the concordance between sTILs, PD-L1 SP142, and 28–8 CPS assays was only moderate, therapeutic responses were observed in discordant cases, indicating that multiple biomarkers may help guide immunotherapy selection in this subtype. Among these, sTILs remain a practical and widely applicable biomarker owing to their simplicity and reliability on standard H&E-stained slides (4, 26).

In our cohort, multivariate analysis revealed that negative lymph node status and lower HER2 single gene expression were independently associated with higher TIL levels (≥ 10%). This aligns with the findings of Caziuc et al., who reported a higher TIL density in node-negative tumors (27). Although TILs are generally regarded as markers of immune activation in more aggressive breast cancer subtypes, our results indicate that within ER+/HER2- tumors, reduced HER2 signaling may contribute to a less immunosuppressive microenvironment and facilitate greater lymphocyte infiltration. Supporting this observation, HER2-negative tumors exhibited slightly but significantly higher TIL densities than that of HER2-low tumors, along with the enrichment of immune-related gene expression signatures (28). This distinct immune landscape may partly result from the activation of HER2 and epidermal growth factor receptor signaling pathways that upregulate programmed death-ligand 1 expression and modulate the tumor immune microenvironment, thereby facilitating immune evasion in HER2-low tumors by creating an immunosuppressive milieu (29). Tumors with lower ERBB2 mRNA expression exhibited significantly higher levels of immune-related chemokines, such as CXCL9, CXCL10, CXCL11, and CXCL13, as observed in our TCGA dataset analysis. CCL5 expression was slightly increased, whereas immune suppression-related chemokines (CCL17 and CCL22) exhibited no significant differences between the groups. This transcriptional pattern may partly explain the increased TIL infiltration observed in HER2-low tumors. HER2-low tumors with higher TILs may thus define an immunologically active subset of ER+/HER2− breast cancer, which could be applied to stratify patients in future clinical trials evaluating immune checkpoint inhibitors in combination with chemotherapy and endocrine therapy. Further studies are warranted to clarify the underlying biological mechanisms and to explore their potential clinical implications.

A primary aspect of our study was the direct comparison between protein and mRNA expression assessed using IHC and Oncotype DX single gene scores, respectively. Oncotype DX gene scores are derived from a standardized, centrally performed RT-PCR assay, offering reproducible and quantitative results that are less susceptible to inter-observer variability than those obtained through IHC (30). Notably, tumors with high TIL levels exhibited significantly lower mRNA expression of ER, PgR, and HER2 when analyzed as continuous variables using Oncotype DX single gene scores. In contrast, when these markers were assessed by IHC using standard binary cut-off values, no significant differences were observed between TIL-high and TIL-low tumors. This discrepancy may reflect the limitations of dichotomized IHC-based classification in detecting subtle but biologically relevant variations in gene expression, indicating that mRNA-based profiling may better delineate immunologically distinct phenotypes.

Our findings support previously reported associations between TILs and Oncotype DX RS in ER-positive and HER2-negative breast cancer (31–34). Because of the widespread clinical use of Oncotype DX, these results indicate that single gene scores, specifically for HER2, can potentially serve as accessible surrogates for immune activation, aiding in the identification of patients who can benefit from immunomodulatory therapies. However, further validation in larger cohorts is required.

This study has several limitations. First, this retrospective single-institution study may be subject to selection bias, particularly related to the indication for Oncotype DX testing, and may therefore limit the generalizability of our findings. In addition, the proportion of tumors with high TIL infiltration was relatively small (12.3%), which could have reduced the statistical power to detect more subtle associations. However, high TIL levels are generally uncommon in ER+/HER2- breast cancer, and the observed distribution in our cohort is consistent with previous reports (24, 31). Despite this limitation, the observed associations were biologically plausible and supported by external validation using TCGA data, suggesting a certain degree of robustness. Second, TILs were assessed solely on H&E-stained sections without immunophenotyping, preventing the assessment of the functional roles of specific lymphocyte subsets (CD8+ cytotoxic T cells vs. regulatory T cells). Third, we did not assess patient outcomes or the prognostic significance of TILs. In addition, tumor-infiltrating lymphocytes are influenced by both tumor-intrinsic characteristics and host-related factors, including comorbidities and concomitant medications (35). However, due to the retrospective nature of this study, detailed and standardized data on these clinical variables were not consistently available, and their potential impact on TIL levels could not be systematically evaluated. Future studies using an integrative approach—including spatial immune profiling, immunophenotyping, and prospective validation—are required to better elucidate the clinical effects of TILs in this setting.

In conclusion, we identified a distinct immunologically active subset within ER+/HER2- breast cancer, characterized by high TIL levels, lymph node-negative status, and low HER2 gene expression, as measured by Oncotype DX. These findings indicate that Oncotype DX single gene scores, specifically HER2, may offer insights beyond recurrence prediction—serving as potential indicators of the tumor immune phenotype. This integrative approach may enhance patient stratification and expand therapeutic strategies for this traditionally less immunogenic breast cancer subtype.

## Data Availability

The fully anonymized minimal dataset underlying the findings of our manuscript has now been deposited in a public repository. The data presented in this study are deposited in the Figshare repository, DOI: 10.6084/m9.figshare.31095262, link: https://doi.org/10.6084/m9.figshare.31095262.
