# Supplementary material for: Association between tumor-infiltrating lymphocytes and oncotype DX estrogen receptor, progesterone receptor, and human epidermal growth factor receptor 2 single gene scores in hormone receptor-positive/HER2-negative breast cancer
Source: Front Oncol. 2026 Jan 27;16:1677929. doi: 10.3389/fonc.2026.1677929 (PMC12888226; doi:10.3389/fonc.2026.1677929)
Supplement: Supplementary file 1 [file Table1.docx]

Supplementary Material

# Supplementary Table

# Supplementary Table 1. TCGA-BRCA sample IDs used in the RNA-seq analysis of ER+/HER2– breast cancer.

| SAMPLE_ID |
| --- |
| TCGA-A2-A0EU-01 |
| TCGA-A2-A0EW-01 |
| TCGA-A2-A0EV-01 |
| TCGA-A2-A0YF-01 |
| TCGA-A2-A0YH-01 |
| TCGA-A8-A06N-01 |
| TCGA-A8-A07L-01 |
| TCGA-A8-A086-01 |
| TCGA-A8-A09C-01 |
| TCGA-A8-A09K-01 |
| TCGA-A8-A0A2-01 |
| TCGA-AN-A0AS-01 |
| TCGA-AN-A0FF-01 |
| TCGA-AN-A0FY-01 |
| TCGA-BH-A0AY-01 |
| TCGA-BH-A0BC-01 |
| TCGA-BH-A0BM-01 |
| TCGA-BH-A0BV-01 |
| TCGA-BH-A0DH-01 |
| TCGA-BH-A0DK-01 |
| TCGA-BH-A0DP-01 |
| TCGA-BH-A0DQ-01 |
| TCGA-BH-A0H0-01 |
| TCGA-BH-A0HK-01 |
| TCGA-A2-A1FV-01 |
| TCGA-A1-A0SE-01 |
| TCGA-GM-A3XG-01 |
| TCGA-GM-A3XN-01 |
| TCGA-A2-A0CL-01 |
| TCGA-A2-A0CV-01 |
| TCGA-A2-A04Y-01 |
| TCGA-A2-A0D4-01 |
| TCGA-A2-A3KD-01 |
| TCGA-A2-A0EX-01 |
| TCGA-A2-A25D-01 |
| TCGA-E2-A14T-01 |
| TCGA-A2-A0T5-01 |
| TCGA-AO-A1KP-01 |
| TCGA-A2-A0ES-01 |
| TCGA-A2-A0D3-01 |
| TCGA-A2-A0EO-01 |
| TCGA-A2-A0SU-01 |
| TCGA-A2-A0T6-01 |
| TCGA-A2-A0T7-01 |
| TCGA-A2-A0YI-01 |
| TCGA-A2-A0YK-01 |
| TCGA-A2-A0YL-01 |
| TCGA-A2-A25C-01 |
| TCGA-A2-A3Y0-01 |
| TCGA-A2-A4RW-01 |
| TCGA-A2-A4RX-01 |
| TCGA-A2-A4RY-01 |
| TCGA-A2-A4S0-01 |
| TCGA-A2-A4S2-01 |
| TCGA-A7-A0CG-01 |
| TCGA-A7-A0CJ-01 |
| TCGA-A7-A0D9-01 |
| TCGA-A7-A0DB-01 |
| TCGA-A7-A13G-01 |
| TCGA-A7-A26E-01 |
| TCGA-A7-A26J-01 |
| TCGA-A7-A3J0-01 |
| TCGA-A7-A4SA-01 |
| TCGA-A7-A5ZW-01 |
| TCGA-A8-A06O-01 |
| TCGA-A8-A06Q-01 |
| TCGA-A8-A06Y-01 |
| TCGA-A8-A06Z-01 |
| TCGA-A8-A079-01 |
| TCGA-A8-A07E-01 |
| TCGA-A8-A07F-01 |
| TCGA-A8-A07G-01 |
| TCGA-A8-A07J-01 |
| TCGA-A8-A081-01 |
| TCGA-A8-A082-01 |
| TCGA-A8-A083-01 |
| TCGA-A8-A084-01 |
| TCGA-A8-A085-01 |
| TCGA-A8-A08I-01 |
| TCGA-A8-A08J-01 |
| TCGA-A8-A08L-01 |
| TCGA-A8-A08O-01 |
| TCGA-A8-A08Z-01 |
| TCGA-A8-A091-01 |
| TCGA-A8-A092-01 |
| TCGA-A8-A093-01 |
| TCGA-A8-A094-01 |
| TCGA-A8-A095-01 |
| TCGA-A8-A096-01 |
| TCGA-A8-A09A-01 |
| TCGA-A8-A09D-01 |
| TCGA-A8-A09M-01 |
| TCGA-A8-A09R-01 |
| TCGA-A8-A09Z-01 |
| TCGA-A8-A0AD-01 |
| TCGA-AC-A2QI-01 |
| TCGA-AC-A3OD-01 |
| TCGA-AC-A62Y-01 |
| TCGA-AN-A0FW-01 |
| TCGA-AO-A1KO-01 |
| TCGA-AQ-A1H3-01 |
| TCGA-AQ-A54O-01 |
| TCGA-AR-A0U2-01 |
| TCGA-AR-A0U3-01 |
| TCGA-AR-A1AK-01 |
| TCGA-AR-A1AL-01 |
| TCGA-AR-A1AO-01 |
| TCGA-AR-A1AS-01 |
| TCGA-AR-A1AV-01 |
| TCGA-AR-A24H-01 |
| TCGA-AR-A24M-01 |
| TCGA-AR-A24R-01 |
| TCGA-AR-A2LK-01 |
| TCGA-AR-A2LN-01 |
| TCGA-AR-A2LQ-01 |
| TCGA-BH-A0BA-01 |
| TCGA-BH-A0BD-01 |
| TCGA-BH-A0BJ-01 |
| TCGA-BH-A0DS-01 |
| TCGA-BH-A0E2-01 |
| TCGA-BH-A0E7-01 |
| TCGA-BH-A0GY-01 |
| TCGA-BH-A0H6-01 |
| TCGA-BH-A0H7-01 |
| TCGA-BH-A0HI-01 |
| TCGA-BH-A0HO-01 |
| TCGA-BH-A0HP-01 |
| TCGA-BH-A0HQ-01 |
| TCGA-BH-A0HU-01 |
| TCGA-C8-A12U-01 |
| TCGA-C8-A12W-01 |
| TCGA-C8-A12X-01 |
| TCGA-C8-A1HG-01 |
| TCGA-C8-A1HI-01 |
| TCGA-C8-A1HM-01 |
| TCGA-C8-A26Z-01 |
| TCGA-C8-A274-01 |
| TCGA-C8-A27A-01 |
| TCGA-D8-A13Y-01 |
| TCGA-D8-A141-01 |
| TCGA-D8-A146-01 |
| TCGA-D8-A1JC-01 |
| TCGA-D8-A1JD-01 |
| TCGA-D8-A1JE-01 |
| TCGA-D8-A1JH-01 |
| TCGA-D8-A1JJ-01 |
| TCGA-D8-A1JP-01 |
| TCGA-D8-A1JS-01 |
| TCGA-D8-A1JU-01 |
| TCGA-D8-A1X6-01 |
| TCGA-D8-A1XB-01 |
| TCGA-D8-A1XC-01 |
| TCGA-D8-A1XF-01 |
| TCGA-D8-A1XM-01 |
| TCGA-D8-A1XO-01 |
| TCGA-D8-A1XR-01 |
| TCGA-D8-A1XU-01 |
| TCGA-D8-A1XZ-01 |
| TCGA-D8-A1Y0-01 |
| TCGA-D8-A1Y1-01 |
| TCGA-D8-A27E-01 |
| TCGA-D8-A27I-01 |
| TCGA-D8-A27K-01 |
| TCGA-D8-A27L-01 |
| TCGA-D8-A27P-01 |
| TCGA-D8-A27T-01 |
| TCGA-D8-A27V-01 |
| TCGA-D8-A4Z1-01 |
| TCGA-E2-A108-01 |
| TCGA-E2-A10C-01 |
| TCGA-E2-A14Q-01 |
| TCGA-E2-A14Z-01 |
| TCGA-E2-A153-01 |
| TCGA-E2-A154-01 |
| TCGA-E2-A15A-01 |
| TCGA-E2-A15F-01 |
| TCGA-E2-A15G-01 |
| TCGA-E2-A15P-01 |
| TCGA-E2-A1B4-01 |
| TCGA-E2-A1IF-01 |
| TCGA-E2-A1IN-01 |
| TCGA-E2-A1L6-01 |
| TCGA-EW-A1PA-01 |
| TCGA-EW-A1PC-01 |
| TCGA-EW-A2FS-01 |
| TCGA-EW-A2FV-01 |
| TCGA-EW-A2FW-01 |
| TCGA-GM-A2DL-01 |
| TCGA-GM-A2DM-01 |
| TCGA-GM-A2DN-01 |
| TCGA-A2-A0CW-01 |
| TCGA-E2-A15K-01 |
| TCGA-E2-A15L-01 |
| TCGA-E2-A15O-01 |
| TCGA-A2-A25B-01 |
| TCGA-A1-A0SJ-01 |
| TCGA-A2-A0CU-01 |
| TCGA-A2-A0ER-01 |
| TCGA-A2-A0SV-01 |
| TCGA-A2-A3KC-01 |
| TCGA-A7-A3IZ-01 |
| TCGA-AO-A1KS-01 |
| TCGA-E2-A1BD-01 |
| TCGA-LL-A50Y-01 |
| TCGA-E2-A15C-01 |
| TCGA-E2-A14S-01 |
| TCGA-E2-A14U-01 |
| TCGA-E2-A15I-01 |
| TCGA-BH-A0GZ-01 |
| TCGA-E2-A10F-01 |
| TCGA-E2-A1IE-01 |
| TCGA-E2-A10E-01 |
| TCGA-A2-A0ET-01 |
| TCGA-A2-A04R-01 |
| TCGA-A2-A04V-01 |
| TCGA-A2-A0T4-01 |
| TCGA-A2-A25A-01 |
| TCGA-A7-A13E-01 |
| TCGA-A7-A13F-01 |
| TCGA-E2-A15D-01 |
| TCGA-E2-A1IO-01 |
| TCGA-E2-A1L8-01 |
| TCGA-E2-A570-01 |
| TCGA-E2-A10B-01 |
| TCGA-E2-A1LA-01 |
| TCGA-A2-A1G0-01 |
| TCGA-A7-A0CD-01 |
| TCGA-AC-A3QP-01 |
| TCGA-B6-A1KI-01 |
| TCGA-E2-A107-01 |
| TCGA-E2-A1IJ-01 |
| TCGA-BH-A0HB-01 |
| TCGA-OL-A66K-01 |
| TCGA-A2-A0CQ-01 |
| TCGA-A2-A0CS-01 |
| TCGA-A2-A0CT-01 |
| TCGA-E2-A15R-01 |
| TCGA-E2-A15T-01 |
| TCGA-A2-A0EN-01 |
| TCGA-A2-A0T3-01 |
| TCGA-AO-A1KT-01 |
| TCGA-E2-A109-01 |
| TCGA-BH-A0H9-01 |
| TCGA-E2-A56Z-01 |
| TCGA-BH-A0HW-01 |
| TCGA-E2-A14O-01 |
| TCGA-BH-A0E1-01 |
| TCGA-LL-A440-01 |
| TCGA-EW-A1PG-01 |
| TCGA-AC-A3TM-01 |
| TCGA-AR-A1AM-01 |
| TCGA-AR-A1AP-01 |
| TCGA-AR-A1AU-01 |
| TCGA-AR-A1AW-01 |
| TCGA-AR-A24K-01 |
| TCGA-AR-A24L-01 |
| TCGA-AR-A24N-01 |
| TCGA-AR-A24P-01 |
| TCGA-AR-A24T-01 |
| TCGA-AR-A2LO-01 |
| TCGA-C8-A130-01 |
| TCGA-C8-A138-01 |
| TCGA-C8-A1HE-01 |
| TCGA-C8-A1HL-01 |
| TCGA-C8-A1HN-01 |
| TCGA-C8-A26W-01 |
| TCGA-D8-A145-01 |
| TCGA-D8-A1JT-01 |
| TCGA-D8-A1XA-01 |
| TCGA-D8-A1XL-01 |
| TCGA-D8-A1XV-01 |
| TCGA-D8-A1Y2-01 |
| TCGA-D8-A1Y3-01 |
| TCGA-D8-A27G-01 |
| TCGA-D8-A27R-01 |
| TCGA-E2-A105-01 |
| TCGA-E2-A15S-01 |
| TCGA-E2-A1IH-01 |
| TCGA-E2-A2P5-01 |
| TCGA-OL-A66H-01 |
| TCGA-A2-A0YD-01 |
| TCGA-LQ-A4E4-01 |
| TCGA-GM-A2DC-01 |
| TCGA-A2-A0CK-01 |
| TCGA-A2-A0CO-01 |
| TCGA-A2-A1FX-01 |
| TCGA-A2-A259-01 |
| TCGA-EW-A1P5-01 |
| TCGA-GM-A2D9-01 |
| TCGA-A2-A0YC-01 |
| TCGA-A2-A04N-01 |
| TCGA-A2-A0CR-01 |
| TCGA-A2-A0SW-01 |
| TCGA-A2-A0EM-01 |
| TCGA-A2-A0EP-01 |
| TCGA-A2-A0YT-01 |
| TCGA-A2-A3XW-01 |
| TCGA-OL-A66J-01 |
| TCGA-AC-A2FF-01 |
| TCGA-A1-A0SB-01 |
| TCGA-A1-A0SD-01 |
| TCGA-A1-A0SF-01 |
| TCGA-A1-A0SI-01 |
| TCGA-A1-A0SQ-01 |
| TCGA-A2-A0CP-01 |
| TCGA-A2-A1FW-01 |
| TCGA-A2-A1FZ-01 |
| TCGA-A2-A1G4-01 |
| TCGA-A2-A4S3-01 |
| TCGA-A7-A3IY-01 |
| TCGA-A7-A3J1-01 |
| TCGA-A7-A3RF-01 |
| TCGA-A7-A426-01 |
| TCGA-A7-A4SB-01 |
| TCGA-A7-A5ZX-01 |
| TCGA-A8-A06P-01 |
| TCGA-A8-A07W-01 |
| TCGA-A8-A09B-01 |
| TCGA-A8-A09Q-01 |
| TCGA-A8-A09T-01 |
| TCGA-A8-A0A1-01 |
| TCGA-A8-A0A4-01 |
| TCGA-A8-A0A9-01 |
| TCGA-AC-A2B8-01 |
| TCGA-AC-A2BM-01 |
| TCGA-AC-A2FG-01 |
| TCGA-AC-A2FK-01 |
| TCGA-AC-A2FO-01 |
| TCGA-AC-A3BB-01 |
| TCGA-AC-A3EH-01 |
| TCGA-AC-A3HN-01 |
| TCGA-AC-A3W7-01 |
| TCGA-AC-A5XS-01 |
| TCGA-AN-A0AM-01 |
| TCGA-AR-A0TY-01 |
| TCGA-AR-A1AH-01 |
| TCGA-AR-A1AJ-01 |
| TCGA-AR-A1AN-01 |
| TCGA-AR-A24O-01 |
| TCGA-AR-A24Q-01 |
| TCGA-AR-A24S-01 |
| TCGA-AR-A2LM-01 |
| TCGA-B6-A1KC-01 |
| TCGA-BH-A0AZ-01 |
| TCGA-BH-A0B0-01 |
| TCGA-BH-A0B5-01 |
| TCGA-BH-A0BF-01 |
| TCGA-BH-A0BO-01 |
| TCGA-BH-A0BP-01 |
| TCGA-BH-A0BR-01 |
| TCGA-BH-A0BS-01 |
| TCGA-BH-A0BZ-01 |
| TCGA-BH-A0C1-01 |
| TCGA-BH-A0C3-01 |
| TCGA-BH-A0DE-01 |
| TCGA-BH-A0DG-01 |
| TCGA-BH-A0DI-01 |
| TCGA-BH-A0DL-01 |
| TCGA-BH-A0DO-01 |
| TCGA-BH-A0DT-01 |
| TCGA-BH-A0DV-01 |
| TCGA-BH-A0DX-01 |
| TCGA-BH-A0E9-01 |
| TCGA-BH-A0EA-01 |
| TCGA-BH-A0EI-01 |
| TCGA-BH-A0H3-01 |
| TCGA-BH-A0H5-01 |
| TCGA-BH-A0HA-01 |
| TCGA-BH-A0HX-01 |
| TCGA-BH-A0W3-01 |
| TCGA-BH-A0W4-01 |
| TCGA-BH-A0W5-01 |
| TCGA-BH-A0W7-01 |
| TCGA-BH-A18F-01 |
| TCGA-BH-A18J-01 |
| TCGA-BH-A18L-01 |
| TCGA-BH-A18N-01 |
| TCGA-BH-A18S-01 |
| TCGA-BH-A1EO-01 |
| TCGA-BH-A1ET-01 |
| TCGA-BH-A1EU-01 |
| TCGA-BH-A1EY-01 |
| TCGA-BH-A1F5-01 |
| TCGA-BH-A1FG-01 |
| TCGA-BH-A201-01 |
| TCGA-BH-A28O-01 |
| TCGA-BH-A28Q-01 |
| TCGA-BH-A42V-01 |
| TCGA-C8-A12M-01 |
| TCGA-C8-A12N-01 |
| TCGA-C8-A12O-01 |
| TCGA-C8-A1HO-01 |
| TCGA-C8-A26V-01 |
| TCGA-C8-A273-01 |
| TCGA-D8-A1J8-01 |
| TCGA-D8-A1JI-01 |
| TCGA-D8-A1JN-01 |
| TCGA-D8-A1X7-01 |
| TCGA-D8-A1X8-01 |
| TCGA-D8-A1X9-01 |
| TCGA-D8-A1XG-01 |
| TCGA-D8-A3Z5-01 |
| TCGA-D8-A3Z6-01 |
| TCGA-E2-A155-01 |
| TCGA-E2-A156-01 |
| TCGA-E2-A15M-01 |
| TCGA-E2-A1B5-01 |
| TCGA-E2-A1BC-01 |
| TCGA-E2-A1IG-01 |
| TCGA-E2-A1IK-01 |
| TCGA-E2-A1IL-01 |
| TCGA-E2-A1IU-01 |
| TCGA-E2-A1L9-01 |
| TCGA-E2-A2P6-01 |
| TCGA-E9-A1NE-01 |
| TCGA-E9-A1NF-01 |
| TCGA-E9-A1NG-01 |
| TCGA-E9-A1NH-01 |
| TCGA-E9-A1NI-01 |
| TCGA-E9-A1R2-01 |
| TCGA-E9-A227-01 |
| TCGA-E9-A22A-01 |
| TCGA-E9-A22B-01 |
| TCGA-E9-A3Q9-01 |
| TCGA-E9-A54X-01 |
| TCGA-EW-A1IX-01 |
| TCGA-EW-A1IY-01 |
| TCGA-EW-A1J1-01 |
| TCGA-EW-A1J2-01 |
| TCGA-EW-A1J6-01 |
| TCGA-EW-A1OX-01 |
| TCGA-EW-A1OY-01 |
| TCGA-EW-A1P0-01 |
| TCGA-EW-A1P3-01 |
| TCGA-EW-A1P6-01 |
| TCGA-EW-A1PE-01 |
| TCGA-EW-A3E8-01 |
| TCGA-EW-A423-01 |
| TCGA-GI-A2C8-01 |
| TCGA-GM-A2DK-01 |
| TCGA-GM-A2DO-01 |
| TCGA-GM-A3NY-01 |
| TCGA-GM-A4E0-01 |
| TCGA-MS-A51U-01 |

TCGA sample identifiers (barcodes) for the 444 ER+/HER2– breast cancer cases analyzed in this study are listed. Cases were selected from the Breast Invasive Carcinoma (TCGA, *Cell* 2015) dataset using cBioPortal (<https://www.cbioportal.org/>), based on clinical annotations. The list provides a reference for reproducibility and enables further analysis by other researchers.
